# Intake of vitamin B12 in relation to vitamin B12 status in groups susceptible to deficiency: a systematic review

**DOI:** 10.29219/fnr.v67.8626

**Published:** 2023-06-30

**Authors:** Linnea Bärebring, Christel Lamberg-Allardt, Birna Thorisdottir, Alfons Ramel, Fredrik Söderlund, Erik Kristoffer Arnesen, Bright I. Nwaru, Jutta Dierkes, Agneta Åkesson

**Affiliations:** 1 Department of Internal Medicine and Clinical Nutrition, Institute of Medicine, Sahlgrenska Academy, University of Gothenburg, Sweden; 2 Department of Food and Nutrition, University of Helsinki, Finland; 3 Faculty of Sociology, Anthropology and Folkloristics and Health Science Institute, University of Iceland, Iceland; 4 Faculty of Food Science and Nutrition, University of Iceland, Iceland; 5 Unit of Cardiovascular and Nutritional Epidemiology, Institute of Environmental Medicine, the Karolinska Institute, Sweden; 6 Department of Nutrition, Institute of Basic Medical Sciences, University of Oslo, Norway; 7 Krefting Research Centre, Institute of Medicine, University of Gothenburg, Sweden; 8 Centre for Nutrition, Department of Clinical Medicine, University of Bergen, Norway; 9 Department of Laboratory Medicine and Pathology, Haukeland University Hospital, Norway

**Keywords:** cobalamin, vitamin B12, holotranscobalamin, methylmalonic acid, homocysteine, dietary guidelines

## Abstract

**Objective:**

To systematically review the evidence for whether habitual or different levels of experimental intake of vitamin B12 from diet and supplements is sufficient to ensure adequate B12 status in groups most susceptible to vitamin B12 deficiency.

**Methods:**

We searched MEDLINE, Embase, Cochrane Central Register of Controlled Trials and Scopus up to 21 May 2021, for intervention studies, prospective cohort studies and case-control studies assessing B12 intake from diet and/or supplements in relation to B12 status (s/p-B12, holotranscobalamin, methylmalonic acid, homocysteine or breastmilk B12). Cross-sectional studies were eligible for studies conducted during pregnancy and lactation. Included populations were children (0–18 years), young adults (18–35 years), pregnant or lactating women, older adults (≥65 years) and vegans or vegetarians. Study selection, data extraction and risk of bias assessment were conducted by two assessors independently. The evidence was synthesized qualitatively and classified according to the World Cancer Research Fund.

**Results:**

The searches yielded 4855 articles of which 89 were assessed in full text and 18 included. Three studies were conducted during pregnancy and three during lactation or infancy – all observational. Eight studies were conducted among older adults; most were interventions among B12-deficient participants. Four studies were eligible for vegetarian and vegans, all interventions. The strength of evidence that habitual B12 intake or an intake in line with the current Nordic recommended intake (RI) is sufficient to ensure adequate status was considered *Limited – no conclusion* for all included populations.

**Conclusion:**

Evidence is insufficient to assess if or which level of B12 intake is sufficient to maintain adequate status for all included populations. Population-based cohort studies and low-to-moderate dose interventions that address this question are highly warranted.

## Popular scientific summary

- This systematic review assesses if intake of B12 is sufficient in groups at increased risk of B12 deficiency and its consequences – children, pregnant and lactating women, young adults, older adults and vegetarians or vegans.
- The results show that there is not enough evidence to say if usual or experimental intake of vitamin B12 is sufficient.
- More high-quality research is needed, especially in light of the current transition towards a more plant-based diet.

## Introduction

Vitamin B_12_ or cobalamin (B12) is an essential nutrient that is vital for human health, primarily as a coenzyme in one-carbon metabolism (1). B12 contributes to blood cell formation, synthesis of DNA, regeneration of the amino acid methionine and the maintenance of myelin that protects the nerve cells among other functions (2). B12 is absorbed in the form found in foods of animal origin, given adequate secretion of hydrochloric acid and the glycoprotein *intrinsic factor* excreted from the parietal cells of the stomach. Once absorbed, a low intake will suffice to maintain adequate status as the nutrient is stored in the body and can be partially reabsorbed by the intestine after excretion with bile (3).

B12 deficiency may lead to megaloblastic anaemia, characterized by large and immature red blood cells (2). Other consequences of deficiency include neurological and cognitive impairment (4). While clinical signs of B12 deficiency may take years to develop, several biological markers of B12 status are available, including serum or plasma concentrations of total vitamin B12/cobalamin (B12), holotranscobalamin (holoTC), methylmalonic acid (MMA) and homocysteine (tHcy) (4). A major risk factor for B12 deficiency is the autoimmune disease pernicious anaemia, which causes destruction of the gastric parietal cells and thereby the intrinsic factor (4). Other population groups are however at increased risk of B12 deficiency, including those who consume limited or no food of animal origin (5), young children with low B12 body stores (6) or older adults with reduced secretion of hydrochloric acid or intrinsic factor (4, 7).

Cross-sectional studies show that B12 status stabilizes at intakes of 4–10 μg/d (8). The Nordic Nutrition Recommendation’s (NNR) 2012 recommended intake (RI) for vitamin B12 (8) in different population groups can be seen in Table 1. There are uncertainties regarding the vitamin B12 content of breastmilk, and the requirement and intake of infants is understudied (9).

**Table 1:** Recommended intake of vitamin B12 according to the Nordic Nutrition Recommendations 2012

Population*	Recommended intake (μg/d)
Infants, 6–11 months	0.5
Infants/children, 12–23 months	0.6
Children, 2–5 years	0.8
Children, 6–9 years	1.3
Adults and children from 10 years	2.0
During lactation	2.6

* No recommended intake for children <6 months.

With the current emphasis on lower meat intake and plant-based diets, it is unclear if the intake of vitamin B12 is sufficient to maintain adequate B12 status in people following different dietary patterns, e.g. vegetarians or vegans. In addition, it is unknown if habitual B12 intake reaches RI and if this is sufficient to ensure adequate status in all age groups, including those most susceptible to deficiency. The aim of this systematic review was to summarize the evidence for whether habitual or different levels of experimental intake of vitamin B12 from diet and supplements are sufficient to ensure adequate B12 status in children, pregnant and lactating women, young adults, older adults, vegetarians and vegans.

## Methods

This systematic review was conducted according to the guidelines for systematic reviews, developed for the 2022 revision of the NNR (10, 11) and preferred reporting for systematic reviews (12). The NNR 2022 is funded by the Nordic Council of Ministers and governmental food and health authorities of Norway, Finland, Sweden, Denmark and Iceland (13). A study protocol was published prior to article selection in database PROSPERO (https://www.crd.york.ac.uk, CRD42021244376).

### Eligibility criteria

The research question was specified by the NNR 2022 Committee and the NNR Systematic Review Centre (i.e. the authors) by defining the population, intervention/exposure, control, timing, study design and setting (PI/ECOTSS). The PI/ECOTSS (Table 2) included six healthy populations relevant for the Nordic setting: (1) children (0–18 years), (2) pregnant women, (3) lactating women, (4) young adults (18–35 years), (5) older adults (≥65 years) and (6) vegetarians, including vegans. The intervention/exposure included both supplemental and dietary intake of vitamin B12, and the comparator was different levels of intake (including placebo). Outcomes were defined as biological markers of vitamin B12 status, either B12, holoTC, MMA, tHcy in plasma or serum, or B12 in breastmilk.

**Table 2:** Population, Intervention/Exposure, Comparator, Outcomes, Timing, Setting and Study designs (PI/ECOTSS) criteria for the papers to be included in the systematic review

Population	Intervention/exposure	Comparator	Outcomes	Timing	Setting	Study design
(1) Children (0–18 years) (2) Young adults (18–35 years) non-pregnant/non-lactating (3) Pregnant women (4) Lactating women (5) Older adults (≥65 years) (6) Vegetarians, including vegans	B12 intake, supplemental and dietary	Different levels of exposure	B12 status: *s/p-B12 *s/p-HOLO-TC *s/p-MMA *s/p-tHcy *Combined indicators *Breastmilk B12 (relevant in infants)	RCTs ≥4 weeks, cohorts ≥12 months	Relevant for the general population in the Nordic and Baltic countries	RCTs, cohort studies, case-control studies, case cohort studies and cross-sectional studies (only for limited periods as pregnancy and lactation)

Eligible study designs were randomized control trials (RCTs), cohort studies or case-control studies. Further, cross-sectional studies were included for pregnant and lactating populations, due to the limited time frame of gestation and lactation. Minimum study duration was 4 weeks for RCTs and 12 months for prospective studies. Intervention studies using intravenous vitamin B12 supplementation or toothpaste enriched with vitamin B12 were excluded. Observational studies were limited to European and North American populations.

### Search strategy

The literature searches were performed by research librarians from the University of Oslo 21 April 2021 in databases MEDLINE, Embase, Cochrane Central Register of Controlled Trials and Scopus. The search strategy (Supplement 1) was developed in collaboration with the authors, and peer reviewed by university librarians from Karolinska Institutet. Reference lists of relevant retrieved articles were also screened to identify additional articles. These searches utilized no restrictions on publication dates or language. Grey literature and unpublished studies were not searched.

### Study selection and data extraction

Screening and selection of studies for inclusion/exclusion was performed independently by two authors (LB and CLA). The screening of titles and abstracts was performed in Rayyan (14). A pilot test was conducted using 10% of the titles and abstracts, in order to harmonize the process. Discrepancies were resolved by discussion with a third author (AÅ). Data from full-text papers included in the systematic review were extracted in standardized extraction forms by authors working independently (EKA, AR, FS).

### Risk of bias assessment

Risk of bias in each included study was assessed by two authors (CLA and BT), working independently. The assessment tools used were for intervention studies Cochrane’s Risk of bias 2.0 (15) and Risk of Bias in Non-randomised Studies of Interventions (16), while ‘Risk of Bias for Nutrition Observational Studies’ (RoB-NObS) (17) was used for prospective observational studies. For cross-sectional studies, the National Heart, Lung, and Blood Institute Quality Assessment Tool for Observational Cohort and Cross-Sectional Studies was used (18). Risk of bias was visualized by using web app Risk-of-bias VISualization (robvis) (19).

### Synthesis and strength of evidence

The evidence was synthesized qualitatively, based on study characteristics, context, strengths and limitations, heterogeneity and relevance. In accordance with the guidelines for systematic reviews, meta-analyses were considered if deemed appropriate to combine/pool the different studies, but only when more than three independent RCTs or five cohort studies exist. Strength of evidence was appraised based on risk of bias, consistency/heterogeneity and precision of the evidence, according to the World Cancer Research Fund’s grading: ‘Convincing’, ‘Probable’, ‘Limited – suggestive’, ‘Limited – no conclusion’, ‘Substantial effects unlikely’ (13).

## Results

**Fig. 1:**
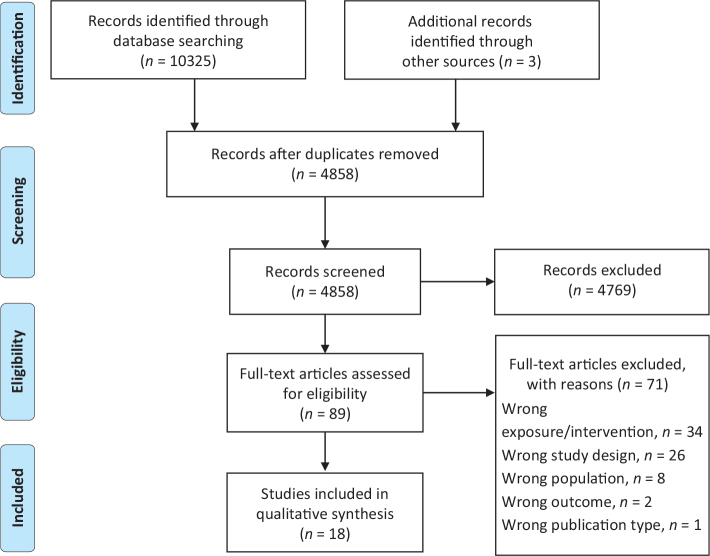
PRISMA 2009 flow diagram.

The searches yielded 4855 unique articles, of which 89 were read in full text and 18 included (Fig. 1). Articles excluded after full text screening are shown in Supplement 2. For two out of the six populations selected for this systematic review (children other than breastfed infants, and young adults), no eligible studies were identified. Due to heterogeneity in types of interventions, exposures and reported outcomes, no meta-analysis was performed. Results are thus limited to qualitative synthesis.

### Pregnant and lactating women and their offspring

#### Study characteristics

Three studies on pregnant women were included (Table 3). In a prospective cohort study from Germany, Koebnick et al. (20) followed (from gestational week 9–12 until the last trimester) 27 lacto-ovo vegetarian pregnant women, 43 low meat eaters (defined as consuming meat <300 g/week and meat products <105 g/week) and 39 omnivore women consuming a Western diet. In another prospective cohort study from Canada, Visentin et al. (21) followed 368 women from gestational week 12–16 until delivery. In a Dutch cross-sectional study, Denissen et al. (22) included 1365 pregnant women in the third trimester.

**Table 3:** Description of studies conducted among pregnant and lactating women and their offspring

Author (year) Country	Population	Design	Treatment/exposures	Dietary assessment methods	Participants (N)	Age at inclusion	Follow-up time	Outcomes
Koebnick 2004 Germany (20)	Pregnant	Prospective cohort	Adhering to lacto-ovo vegetarian diet, low meat diet or Western diet	4-day semi-quantitative food record	N = 109 (27 lacto-ovo vegetarians, 43 low meat eaters, 39 controls)	Age: 29–31 years Gestational week: 9–12	From weeks 9–12 through 36–38 of gestation	s-B12, s-holo-TC, p-tHcy
Visentin 2016 Canada (21)	Pregnant	Prospective cohort	Dietary vitamin B12 intake	Block FFQ	N = 368 included (N = 364 at baseline, N = 309 at endpoint)	Age: 32 years Gestational week: 12–16	From week 12 to 16 until delivery	s-B12, p-MMA, p-tHcy
Denissen 2019 Netherlands (22)	Pregnant	Cross-sectional	Vitamin B12 intake	Semi-quantitative FFQ	N = 1266	Age: 32.6 years Gestational week: Third trimester	N/A	p-B12, p-holoTC, p-MMA
Greibe 2013 Denmark (23)	Lactation	Prospective cohort	Vitamin B12 intake from breastmilk	N/A	60 Mother-child pairs	Mothers: Median age 30 years, 2 weeks postpartum Children: Birth	9 months	B12 in breast milk, p-B12, p-holoTC, p-MMA
Henjum 2020 Norway (24)	Lactation	Cross-sectional	Maternal vitamin B12 intake from diet and supplement	FFQ	N = 193 (175 analysed)	Age: 32 years, 0–6 months postpartum	N/A	Breastmilk B12
Hay 2008 Norway (25)	Lactation and infants	Prospective cohort	Vitamin B12 from diet and supplements	Questionnaire on intake of breastmilk or formula at 6 months, semi-quantitative FFQ and 7-day weighed food record at 12 months	N = 364 (249 at 12 months)	Mothers: Mean age 29.9 years Children: Birth	From birth until 2 years age	s-B12, s-holoTC, s-MMA, s-tHcy

Two studies on lactating women were included (Table 3). In a Danish prospective cohort study, Greibe et al. (23) included 60 mother-child pairs and studied associations both between maternal B12 intake with maternal B12 status and B12 content of breastmilk in addition to associations with infant B12 status. In a cross-sectional study from Norway, Henjum et al. (24) included 193 women 0–6 months postpartum and assessed B12 intake from supplements and diet as well as B12 in breastmilk.

One prospective cohort study from Norway by Hay et al. (25) included infant data on both B12 intake and status at 6, 9 and 12 months.

### Intake of B12 in relation to status

Among the studies conducted during pregnancy, Koebnick et al. (20) found that median dietary intake of B12 was 2.5 μg/d, 3.8 μg/d and 5.3 μg/d among lacto-ovo vegetarians, low meat eaters and omnivore pregnant women, respectively (Table 4). The corresponding proportion of B12 supplement users was 32, 28 and 21%. The odds of B12 deficiency (<100–130 pmol/L) were almost 4 times higher among ovo-lacto vegetarians and almost 2 times higher among low meat eaters, compared to omnivores. Visentin et al. (21) found that prevalence of B12 deficiency (s-B12 <148 pmol/L) was 17% in early pregnancy and 38% in mid-to-late pregnancy. For every 10-μg increment in maternal total vitamin B12 intake, s-B12 increased by 1.04 pmol/L in both early pregnancy and mid-to-late pregnancy in repeated cross-sectional analyses. Corresponding decreases were observed for tHcy and MMA. In the cross-sectional study by Denissen et al. (22), the mean vitamin B12 intake for all pregnant women was 5.0 μg/day. The corresponding results for subgroups were for omnivores 5.1 μg/day, pescatarians 4.3 μg/day, vegetarians 3.5 μg/day (self-defined) and lacto-ovo vegetarians 2.3 μg/day (defined by researchers based on FFQ data). The authors found dose-response associations between total dietary vitamin B12 intake with p-B12, holoTC and MMA. The odds of B12 deficiency were lower in the second (5 μg/day) and third tertiles (9.1 μg/day) of B12 intake, compared with the first tertile (3.2 μg/day). The analysis showed that a vitamin B12 intake of ≥4.2 μg/day was associated with ~90% lower odds of deficiency compared to lower intake.

**Table 4:** Summary of results and overall risk of bias for studies conducted during pregnancy, lactation or infancy

Author (year) Population	Outcome, definition	Vitamin B12 intake	Results	Effect estimates (final models)	Overall risk of bias
Koebnick 2004 Pregnancy (20)	**Low B12 status (pmol/L):** s-B12: First trimester <130 Second trimester <120 Third trimester <100 **Elevated p-tHcy μmol/L**: First trimester: >9 Second to third trimester: >7.8	**Dietary intake median (IQR) μg/d:** Lacto-ovo vegetarians: 2.5 (1.3–3.8) (*P* < 0.001 vs. controls) Low meat eaters (<405 g/week): 3.8 (3.0–4.9) (*P* < 0.001 vs. controls) Western diet controls: 5.3 (4.3–6.3) **Supplement use:** Lacto-ovo vegetarians: 32.1% (*P* = 0.889 vs. controls) Low meat eaters 27.9% (*P* = 0.559 vs. controls) Western diet controls: 20.5%	**Lacto-ovo vegetarians:** ↓ sB12 ↓holo-TC ↑ tHcy ↑ odds of low B12 status 39% low s-B12 **Low meat eaters:** ↓ sB12 ↑ tHcy (*P* = 0.061) ↑ odds of low B12 status 9% low s-B12 Optimal daily B12 intake during pregnancy should be >3 μg vitamin B12	**Risk of low B12 status:** OR (95% CI) = 3.9 (1.9–6.1) for lacto-ovo vegetarians compared to controls OR (95% CI) = 1.8 (1.0–3.9) for low meat eaters compared to controls	Serious
Visentin 2016 Pregnancy (21)	**B12 deficiency (pmol/L):** s-B12 <148 **Marginal B12 deficiency (pmol/L):** s-B12: 148–220 **Elevated p-tHcy (μmol/L):** >13 **Elevated p-MMA (nmol/L):** <271	**Dietary intake mean (SD) μg/d:** Weeks 0–16: 4.7 (3.1) Weeks 23–37: 4.6 (2.6) **Supplement intake median (IQR) μg/d:** Weeks 0–16: 2.6 (2.6–10.0) Weeks 23–37: -	**Total vitamin B12 intake in early pregnancy**: ↑ s-B12 (16.9% deficient, 35% marginal) ↓tHcy (0% elevated) ↓MMA (1.9% elevated) **Total vitamin B12 intake in mid-to late pregnancy:** ↑s-B12 (38.2% deficient, 42.9% marginal) ↓tHcy (0% elevated) ↓ (5.3% elevated) **Supplement use:** ↑s-B12	s-B12 decreased by 23% during pregnancy (*P* = 0.005) **Per 10 μg increase in maternal total vitamin B12 intake:** In early pregnancy, β (95% CI): s-B12 (pmol/L): 1.04 (1.02–1.06) tHcy (μmol/L): -0.04 (-0.08–0.006) MMA (nmol/L): 1.00 (0.99–1.02) Mid-to-late pregnancy, β (95% CI): s-B12 (pmol/L): 1.04 (1.02–1.06) tHcy (μmol/L): 0.03 (-0.04–0.11) MMA (nmol/L): 0.96 (0.93–0.98)	Moderate
Denissen 2019 Pregnancy (22)	Vitamin B-12 deficiency: holoTC <35 pmol/L and MMA >0.45 μmol/L	**Dietary intake mean (95% CI) ug/day:** All: 5.0 (3.8. 6.5) Omnivores: 5.1 (3.9. 6.6) Vegetarians: 3.5 (2.4. 4.7) Pescetarians: 4.3 (3.3. 6.1) Lacto-ovo vegetarians: 2.3 (1.9. 2.9)	Dose-response associations between of dietary vitamin B-12 intake and p-B12, holoTC and MMA. B12 intake of ≥4.2 μg/d associated with ~90% reduced odds of deficiency compared to a lower intake.	**% Difference (95% CI), per 1 μg increment in B12 intake:** p- B12: 1.0 (0.9–2.0) p-holoTC: 3.0 (2.0–3.0) p-MMA: −2 (−3.0 to −1.0) **Deficiency, OR (95% CI):** Tertile 2 vs. 1 (5.0 vs. 3.2 ug): 0.07 (0.02–0.33) Tertile 3 vs. 1 (9.1 vs. 3.2 ug): 0.1 (0.03–0.37)	Study quality: moderate
Greibe 2013 Lactation and infancy (23)	Continuous	**Mothers:** Supplement intake: 2 weeks: 79% 4 months: 67% 9 months: 50% **Infants:** Estimated intake from breastmilk**:** 2 weeks: 0.7 μg/d 4 months: 0.3 μg/d 9 months: -	↑ Maternal B12 status = ↑ breastmilk B12↑ Breastmilk B12 = ↑ infant B12 status at 4 months B12 content of breastmilk decreased over time **Exclusive breastfeeding vs. not exclusive breastfeeding at 4 months:** ↓ B12 ↓ holoTC	**Maternal and infant p-B12 concentration:** 2 weeks: r = 0.52 (*P* = 0.0001) 4 months: r = 0.47 (*P* = 0.0001) 9 months: r = 0.29 (*P* = 0.03) **Median (range) B12 content of hind milk, pmol/L:** 2 weeks: 760 (210–1880) 4 months: 290 (140–690) 9 months: 440 (160–1940) (all significantly different) **Breastmilk B12 at 4 months postpartum:** infant p-B12: r = 0.58 (*P* = 0.005)	Low
Henjum 2020 Lactation (24)	Continuous	**Mothers:** **Vitamin B12 intake (μg/d):** Diet: 4.1 Diet + supplements: 5.0 Supplement use: 34%	Breastmilk B12 content not associated with maternal B12 intake **Infant, B12 intake estimated from breastmilk (exclusive breastfeeding non-supplemented mothers), μg/d:**1 months: 0.47 2 months: 0.33 3 months: 0.25 4 months: 0.28 5 months: 0.31 6 months: 0.29	**Breastmilk B12 concentration:** B12 supplement users vs. non-users mean (SD): 340 (179) vs. 320 (169) pmol/L, *P* = 0.46 Dietary B12 intake, β (95% CI): 3.8 (-7.0-14.6), *P* = 0.49 **Content of breastmilk decreased over time content,** β **(95% CI):** Per week: −5.0 (−9.7 to −0.2), *P* = 0.04	Study quality: low
Hay 2008 Lactation and infancy (25)	Continuous	**B12 intake at 12 months, geometric mean (95% CI) μ/d**: Breastfed: 1.4 (1.3–1.6) Non-breastfed: 2.4 (2.1–2.6) (*P* < 0.001)	**Exclusive breastfeeding infants at 6 months:** ↓ sB12 (vs. non-breastfed) ↓holo-TC (vs. non-breastfed) **Breastfeeding infants at 12 months:** ↓ sB12 (vs. non-breastfed) ↓holo-TC (vs. non-breastfed) ↑ tHcy (vs. non-breastfed) ↑MMA (vs. non-breastfed) **Introduction of formula and/or solids at 6 and 12 months:** ↑ sB12 ↑holo-TC ↓ tHcy ↓ MMA	**6 mo s-B12 (mean [95% CI]) pmol/L**: Exclusive breastfed: 242 (202–289) Non-breastfed**:** 365 (328–408) Breastfed + solids**:** 244 (226–264) Breastfed + solids and breastmilk substitutes: 249 (226–274) **Correlation between B12 intake from complementary foods and B12 status at 12 months:** sB12: r = 0.15 (*P* = 0.03) holoTC: r = 0.25 (*P* = 0.001) **Correlation between no breastfeedings/d and B12 status at 12 months**: sB12: r = -0.25 (*P* < 0.001) holoTC: r = -0.33 (*P* < 0.001) **12 months B12 (mean [95% CI]) pmol/L**: Breastfed + solids**:** 288 (242–342) Non-breastfed**:** 397 (372–424) Breastfed + solids and breastmilk substitutes: 343 (319–369)	Low

Among the studies conducted during lactation, Greibe et al. (23) found that maternal B12 status was significantly correlated with B12 content of breastmilk at 4 months postpartum. Consequently, B12 content of breastmilk at 4 months postpartum correlated with infant p-B12, while there was no correlation between breastmilk B12 and holoTC or MMA in the children at any time point. Exclusively breastfed infants at 4 months had lower p-B12 and holoTC concentrations than infants not exclusively breastfed. Henjum et al. (16) found no significant correlation between maternal B12 intake (from either supplements or diet) and breastmilk B12 content. However, breastmilk B12 content was found to decrease over time during the 6 month period.

Hay et al. (17) found that both at 6 and 12 months, all biomarkers of infant B12 status were affected by feeding pattern. At 6 months, B12 status was lower among breastfed infants (with or without complementary feeding) compared to non-breastfed infants. At 12 months, those partially breastfed still had lower B12 status and intake (excluding B12 content of breastmilk) than non-breastfed children.

### Risk of bias and strength of evidence

**Fig. 2:**
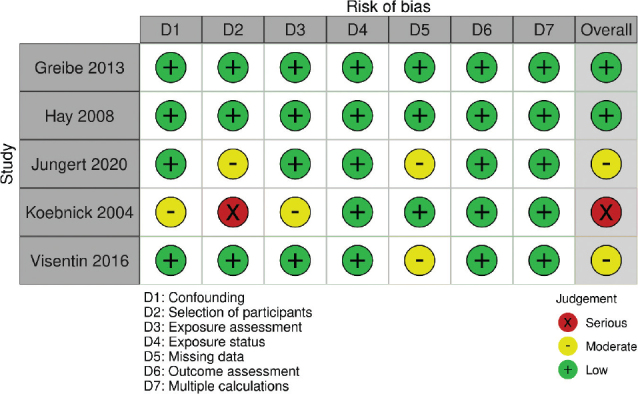
Risk of bias per domain and overall, for all included cohort studies.

Overall risk of bias in studies in pregnant women was regarded as *serious* for Koebnick et al. (20) and *moderate* for Visenitin et al. (21) (Fig. 2). For the cross-sectional study by Denissen et al. (22), we only assessed study quality, which was regarded as *moderate*. The overall risk of bias in studies of lactating women and their offspring was regarded as *low* for Greibe et al. (23) and *low* for Hay et al. (25). The general quality of the cross-sectional studies was regarded as *low* for Henjum et al. (24).

The strength of evidence that habitual B12 intake or an intake in line with the current Nordic RI is sufficient to ensure adequate status during pregnancy, lactation or infancy is considered *Limited – no conclusion*. There was *Limited – suggestive* evidence that breastfed infants (exclusive and/or any breastfeeding) have lower B12 status than non-breastfed infants at 4–6 months of age. Evidence was however *Limited – no conclusion* for that breastfed infants were at higher risk of B12 deficiency. Overall, the evidence was regarded as limited due to scarcity of data and a lack of eligible studies.

## Older adults (≥65 years)

### Study characteristics

Eight studies on older adults were included – five RCTs, conducted in Australia (26), the Netherlands (27, 28), Switzerland (29) and the United Kingdom (30), one nonrandomized intervention study conducted in the United States (31), one RCT conducted in the United States (32) and one prospective cohort study conducted in Germany (33) (Table 5). Sample sizes in intervention studies ranged from 23 to 149 subjects with a mean/median age of 75–82 years. Study duration ranged from 4 to 18 weeks. Supplement doses ranged from 2.5 to 1000 μg/day. One study gave capsules or vitamin B12-fortified milk (28), while the others used oral supplements. The prospective cohort by Jungert et al. (33) studied 332 adults ≥60 years, for 12 years.

**Table 5:** Description of studies conducted among older adults (≥65 years)

Author year Country	Design	B12 dose or exposure	Dietary assessment methods	Participants N	Age at inclusion	Follow-up time	Outcome	Baseline B12 status
Seal 2002 Australia (26)	RCT	10, 50 μg/d vs. placebo	N/A	31	Mean 81.4 years	4 weeks	s-B12, Hcy	s-B12 100–150 pmol/L
Eussen 2005 Netherlands (27)	RCT	2.5, 100, 250, 500 and 1000 μg/d	N/A	120	Mean 80 years	16 weeks	s-B12, p-MMA, p-tHcy, p-holoTC	s-B12 100–300 pmol/L
Dhonokushe-Rutten 2005 Netherlands (28)	RCT	Milk: 7000 μg B12/L vs. placebo Capsules: 1000 μg/d vs. placebo	N/A	Milk: 20 Capsules: 23 Placebo milk: 21 Placebo capsules: 14	Mean 81–82 years	12 weeks	s-B12, p-MMA, p-tHcy	S-B12 100–300 pmol/L and p-MMA ≥0.30 μmol/L
Favrat 2011 Switzerland (29)	Pragmatic RCT, parallel	1000 μg/d vs. placebo	N/A	50	Median 75.5 years	1 month	s-B12, s-MMA, s-tHcy	s- B12 125-200 pM/L
Hill 2013 UK (30)	RCT, parallel	500, 100 and 10 μg/d vs. placebo	N/A	100	Median 71 years	2 months	p-B12, p-MMA, p-tHcy, s-holoTC	p-B12 <250 pmol/L, MMA/mmol creatinine >1.5
Rajan 2002 USA (31)	Nonrandomized intervention	25 μg/d for 6 weeks, 100 μg/d for 6 weeks, 1000 μg/d for 6 weeks	N/A	40	65 years or older, mean age 79 years	18 weeks	s-MMA, s-tHcy	s-B12 <221 pmol/L and s-MMA >271 nmol/L
Stabler 2006 USA (32)	RCT	0, 25 or 100 μg/d	N/A	149 (45 with elevated MMA)	Mean 76.3 years	3 months	s-B12, s-MMA, s-tHcy	s-MMA <271 (all >271 offered 1000 μg/d)
Jungert 2020 Germany (33)	Prospective cohort	Cobalamin from diet and supplements	3-day dietary record	332	Age of at least 60 years (median 68 years)	12 years	s-B12	N/A

### Intake of B12 in relation to status

Among studies with deficient or borderline-deficient subjects, Seal et al. (26) found that B12 supplementation of 50 μg/d, but not 10 μg/d, improved B12 status (Table 6). Eussen et al. (27) found that supplemental B12 doses of 2.5, 100, 250, 500 and 1000 μg/d increased s-B12 and holoTC while MMA and tHcy decreased, all in a dose-response manner. Favrat et al. (29) found that B12 supplementation of 1000 μg/d significantly increased s-B12 and decreased MMA and tHcy in comparison to placebo. Hill et al. (30) found that 10 μg/d elicited improvement in B12 status but 500 μg/d was required to normalize p-B12 and holoTC in 90% of participants during the 2-month intervention. Dhonokushe-Rutten et al. (28) found that fortified milk increased s-B12 and decreased MMA and tHcy similar to capsule supplements. Rajan et al. (31) showed that 25 μg/d for 6 weeks was sufficient to normalize MMA in 2 out of 20 subjects while 100 μg/d normalized MMA in an additional 5 out of 20 subjects. Most did not normalize their elevated serum MMA levels until the 1000 μg dose.

**Table 6:** Summary of results and overall risk of bias for studies conducted during among older adults

Author, yearStudy design	Results B12, pmol/L	Results MMA, μmol/L	Hcy, μmol/L	Holo-TC, pmol/L	Risk of bias
Seal 2002 RCT (26)	**Baseline mean (SD):** Placebo: 137.9 (24.0) 10 μg: 140.3 (26.6) 50 μg: 162.9 (39.2) **Endpoint**: Placebo: +11.7(24.5)% 10 μg: +40.2 (34.4)% 50 μg: +51.7 (47.1)%	-	**Baseline:** Placebo: 26.4 μmol/L 10 ug: 28.4 (SD 9.6) 50 ug: 20.8 (SD 5.9) **Endpoint:** Placebo: −3.6 (24.6)% 10 μg: −10.1 (27.1)% 50 μg: −15.6 (18.0)%	-	Low
Eussen 2005 RCT (27)	**Baseline median (IQR):** 208 (87) **Endpoint (16 weeks) median (IQR):** 2.5 μg: 290 (119) 100 μg: 279 (184) 250 μg: 347 (188) 500 μg: 404 (293) 1000 μg: 574 (418)	**Baseline median (IRQ):** 0.33 (0.16) **Endpoint (16 weeks) median (IQR):** 2.5 μg**:** 0.28 (0.07) 100 μg: 0.30 (0.07) 250 μg: 0.28 (0.11) 500 μg: 0.26 (0.03) 1000 μg: 0.25 (0.04)	**Baseline median (IRQ):** 14.5 (5.7) **Endpoint (16 weeks) median (IQR):** 2.5 μg: 14.0 (6.5) 100 μg: 13.6 (4.5) 250 μg: 13.8 (6.1) 500 μg: 13.1 (5.4) 1000 μg: 10.4 (5.1)	**Baseline median (IRQ):** 47 (35) **Endpoint (16 weeks) median (IQR):** 2.5 μg: 63 (40) 100 μg: 77 (38) 250 μg: 94 (67) 500 μg: 106 (48) 1000 μg: 132 (43)	Low
Dhonokushe-Rutten 2005 RCT (28)	**Baseline mean (SD):** B12 milk: 182 (60) B12 capsule: 171 (51) Placebo milk: 195 (55) Placebo capsule: 206 (64) **Endpoint mean (SD):** B12 milk: 432 (134) B12 capsule: 453 (165) Placebo milk: 207 (68) Placebo capsule: 206 (65)	**Baseline median (p5–p95):** B12 milk: 0.39 (0.22–0.96) B12 capsule: 0.38 (0.25–3.24) Placebo milk: 0.38 (0.25–1.14) Placebo capsule: 0.38 (0.25–1.14) **Endpoint median (p5–p95):** B12 milk: 0.22 (0.15–0.33) B12 capsule: 0.23 (0.14–0.60) Placebo milk: 0.44 (0.24–1.00) Placebo capsule: 0.34 (0.25–1.07)	**Baseline median (p5–p95):** B12 milk: 16.0 (8.3–24.7) B12 capsule: 17.6 (10.1–26.5) Placebo milk: ? Placebo capsule: 14.3 (9.8–25.0) **Endpoint median (p5–p95):** B12 milk: 11.9 (8.1–18.6) B12 capsule: 13.4 (10.4–23.2) Placebo milk: 15.1 (7.7–32.2) Placebo capsule: 14.2 (10.0–21.6)		Low
Favrat 2011 RCT (29)	**Baseline mean (SD):** 1000 μg: 164 (24) Placebo: 154 (20) **Endpoint (1 month) mean (SD):** 1000 μg: 263.4 (89.8) Placebo: 154.5 (41.1)	**Baseline mean (SD):** 1000 μg: 0.43 (0.25) Placebo: 0.41 (0.24) **Endpoint (1 month) mean (SD):** 1000 μg: 0.23 (0.08) Placebo: 0.37 (0.14)	**Baseline mean (SD):** 1000 μg: 18.3 (6.6) Placebo: 15.0 (5.3) **Endpoint (1 month) mean (SD):** 1000 μg: 16.5 (6.1) Placebo: 13.9 (4.3)	-	Low
Hill 2013 RCT (30)	**Baseline median (range):** Placebo: 188 (122–249) 10 μg: 202 (115–239) 100 μg: 216 (127–249) 500 μg: 183 (107–245) Endpoint (56 days) mean*: Placebo: 185 10 μg: 246.5 100 μg: 274 500 μg: 342	**Baseline mean:** Placebo: never smokers: not stated Placebo: ex-smokers: not stated 10 μg: never-smokers: 0.34 10 μg: ex-smokers: 0.39 μmol/L 100 μg: never-smokers: 0.33 100 μg: ex-smokers: 0.40 500 μg: never-smokers: 0.23 500 μg: ex-smokers: 0.34 Endpoint (56 days) mean*: Placebo never-smokers: 0.39 Placebo ex-smokers: 0.60 10 μg never-smokers: 0.34 10 μg ex-smokers: 0.39 100 μg never-smokers: 0.33 100 μg ex-smokers: 0.40 500 μg never-smokers: 0.23 500 μg ex-smokers: 0.34	**Baseline:** not stated **Endpoint:** Placebo: 14.6 (0.31) 10 μg: 14.0 (0.30) 100 μg: 15.8 (0.31) 500 μg: 15.1 (0.30)	**Baseline:** not stated **Endpoint mean:** Placebo: 40 10 μg: 65 100 μg: 66 500 μg: 97	Low
Rajan 2002 RCT (31)	-	**Baseline mean (SD)**: 0.581 (0.351) Endpoint (6 weeks) mean*: 25 μg/d: ≈0.396100 μg/d ≈0.3741000 μg/d ≈0.188	Values not stated	-	Low
Stabler 2006 RCT (32)	**Baseline mean (SD):** Placebo: not stated 25 μg: not stated 100 μg: 364 (123) **Endpoint mean (SD):** Placebo: not stated 25 μg: not stated 100 μg: 424 (147)	**Baseline mean (SD):** Placebo: not stated 25 μg: not stated 100 μg: 0.199 (SD 0.37) **Endpoint mean (SD):** Placebo: not stated 25 μg: not stated 100 μg: 0.187 (0.52)	**Baseline mean (SD):** Placebo: not stated 25 μg: not stated 100 μg: 9.0 (SD 2.0) **Endpoint mean (SD):** Placebo: not stated 25 μg: not stated 100 μg: 8.2 (2.2)	-	High
Jungert 2020 Cohort (33)	**s-B12. median (Q1-Q3)**: All: 266.8 (207.6–376.8) Supplement users: 296.8 Nonsupplement users: 259.8 **B12 intake: Median (Q1–Q3)**: All: 5.3 μg/d **Association with s-B12: Parameter estimate (95% CI):** Dietary B12 intake: 2.16 (-4.25–8.58). *P* = 1.000 Supplemental intake: 96.86 (50.66–143.06). *P* < 0.001	**-**	-	-	Moderate

* Estimated from figure by Graphreader.com.

Among nondeficient older participants, Stabler et al. (32) reported no between group differences in s-B12 concentration (0, 25 or 100 μg/day) in the RCT study. However, MMA increased in the placebo group, while tHcy decreased in the 100 μg group. Stabler et al. (32) reported that 30% of subjects had low B12 status based on elevated MMA (>271 nmol/L). These were not included in the RCT.

In the cohort study by Jungert et al. (33), s-B12 was generally adequate (median 267 pmol/L, Table 6). In addition, 9% of females and 16% of males had B12 deficiency (s-B12 ≤ 148 pmol/L). Median B12 intake was 5.3 μg/d and 28% of females and 15% of males had an intake below 4.0 μg/d. In longitudinal analysis, s-B12 was associated with supplemental but not dietary B12 intake. Supplement users had ~97 pmol/L higher s-B12 than nonusers.

### Risk of bias and strength of evidence

**Fig. 3:**
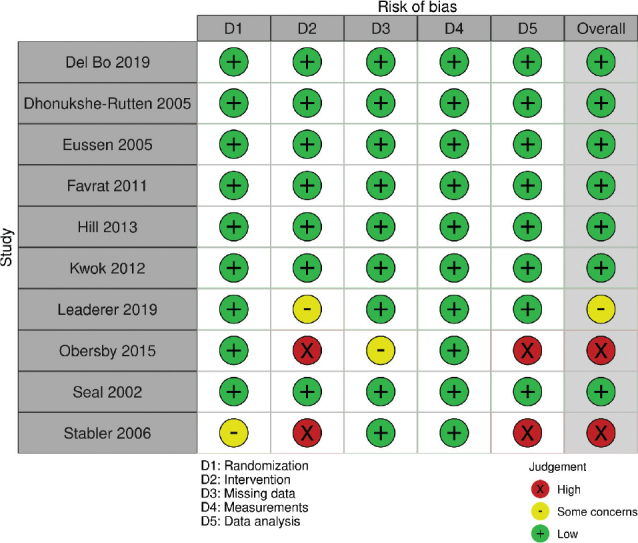
Risk of bias per domain and overall, for all included randomized control trial (RCT) studies.

In the interventions, risk of bias was considered *low* for Seal et al. (26), Eussen et al. (27), Dhonokushe-Rutten et al. (28), Farvat et al. (29) and Hill et al. (30) and *high* for Stabler et al. (32) (Fig. 3). In the nonrandomized intervention by Rajan et al. (31), risk of bias was considered *low* overall and for all included domains. Risk of bias in the cohort by Jungert et al. (33) was considered *moderate* (Fig. 2).

The strength of evidence that habitual B12 intake or an intake in line with the current Nordic RI is sufficient to ensure adequate status among older adults is considered *Limited – no conclusion* due to a lack of eligible population-based prospective studies and low-dose intervention studies. The strength of evidence that higher experimental doses of B12 result in higher biomarkers of status in older adults with B12 deficiency is considered *Convincing* but no optimal intake level could be defined.

## Vegetarians including vegans

### Study characteristics

Four RCTs conducted among vegetarians and vegans were included (34–37) (Table 7). The studies were conducted in Hong Kong (34), the United Kingdom (35), Germany (36) and Italy (37). All studies were conducted among adults, three among practising vegetarians or vegans (34, 35, 37), while one studied effects of a vegan diet (36).

**Table 7:** Description of studies conducted among vegetarians, including vegans

Author year Country	Design	B12 dose or exposure	Participants N	Age at inclusion	Follow-up time	Type of outcome	Population at baseline
Kwok 2012 Hong Kong (34)	RCT, crossover	500 μg/d vs. placebo	50	Mean 45 years	12 weeks, 10-week washout before crossover and additional 24 weeks	s-B12, p-tHcy	Vegetarian for ≥6 years, no regular supplement use
Obersby 2015 UK (35)	RCT, parallel	1000 g every other day vs. placebo	49	Mean ≈ 47 years	16 weeks	p-tHcy	Vegetarian for >3 years, p-tHcy ≥10 μmol/L
Lederer 2019 Germany (36)	RCT, parallel	Strict vegan diet vs. meat-rich diet (>150 g meat/d)	53	Mean 31.5 years	4 weeks	s-B12, s-holo-TC, s-MMA, p-tHcy	Healthy omnivore subjects, BMI 21–30 kg/m^2^
Del Bo 2019 Italy (37)	RCT, parallel	350 μg/week vs. 2000 μg/week	40	Mean 42.5 years	12 weeks	s-B12, s-holoTC, s-MMA, p-tHcy	Vegans and vegetarians, s-B12 <220 pmol/L

Kwok et al. (34) compared 500 μg/day to placebo in a cross-over study among 50 long-term vegetarians (≥6 years) with 12 weeks intervention periods separated by 10 weeks washout. Obersby et al. (35) compared 500 μg/day to placebo for 16 weeks in a parallel design, among 49 long-term vegetarians (>3 years). Del Bo et al. (37) compared a low dose of 50 μg/day (350 μg/week) of B12 to a high dose of 2000 μg/week for 12 weeks in a parallel study among 40 practicing vegetarians with B12 deficiency. Lederer et al. (36) compared a strict vegan diet intervention to a meat-rich control diet for 4 weeks, among 53 omnivores.

### Intake of B12 in relation to status

Kwok et al. (34) found that 500 μg/d of B12 raised s-B12 to 380 pmol/L and lowered tHcy to 11.3 (Table 8). Obersby et al. (35) found that, in intention-to-treat (ITT) analysis, tHcy decreased from 14.7 to 9.1 μmol/L in the group supplemented with 500 μg/d. Del Bo et al. (37) found that both 2000 μg/week and 50 μg/day (350 μg/week) improved B12 status, reflected by higher s-B12 and holoTC and lower MMA and tHcy. Only s-B12 differed significantly between the two doses at 90 days follow-up. Lederer et al. (36) found that after 4 weeks of vegan diet, s-B12 and holoTC had decreased significantly compared to the meat-rich control diet. MMA and tHcy were not significantly different.

**Table 8:** Summary of results and overall risk of bias for studies conducted during among vegetarians, including vegans

Author, year Study design	Results B12, pmol/L	Results MMA μmol/L	Hcy, μmol/L	Holo-TC, pmol/L	Risk of bias
Kwok 2012 RCT (34)	**Baseline mean (SD):** 134 (126) **Endpoint (12 week) mean (SD):** 500 μg: 379.6 (206.2) Placebo: 185.7 (145.4)		**Baseline mean (SD):** 16.7 (11.0) **Endpoint (12 week) mean (SD):** 500 μg: 11.3 (6.0) Placebo: 13.1 (5.0)	-	Low
Obersby 2015 RCT (35)	-	-	**Baseline mean (SD):** ITT 500 μg/d: 14.7 (3.7) Placebo: 14.1 (2.8) Completers: 500 μg/d: 15.5 (3.7) Placebo: 13.7 (2.6) **Endpoint mean (SD):** ITT 500 μg/d: 9.1 (3.1) Placebo: 12.9 (4.5) Completers: 500 μg/d: 8.4 (3.1) Placebo: 12.5 (4.5)	-	High
Lederer 2019 RCT (36)	Baseline mean (SD)**: Vegan: 161.5 (49.4) Meat: 174.1 (70.9) Endpoint mean (SD)**: Vegan: 131.8 (41.9) Meat: 174.4 (63.6)	**Baseline mean (SD) nmol/L:** Vegan: 214.5 (129.6) Meat: 220.0 (121.0) **Endpoint mean (SD) nmol/L:** Vegan: 277.8 (330.0) Meat: 213.1 (182.2)	Values not stated	**Baseline mean (SD):** Vegan: 67.3 (23.5) Meat: 69.7 (29.7) **Endpoint mean (SD):** Vegan: 43.6 (20.0) Meat: 64.4 (28.7)	Some concerns
Del Bo 2019 RCT (37)	**Baseline mean (SD):** 350 μg/w: 146 (36) 2000 μg/w: 131 (56) Endpoint (90 d) mean*: 350 μg/w: 173 2000 μg/w: 200	**Baseline mean**: 350 μg/w: 1.1 2000 μg/w: 1.3 Endpoint (90 d) mean*: 350 μg/w: 0.3 2000 μg/w: 0.4	**Baseline mean**: 350 μg/w: 16 2000 μg/w: 18 Endpoint (90 d) mean*: 350 μg/w: 8.0 2000 μg/w: 8.5	**Baseline mean:** 350 μg/w: 57 2000 μg/w: 45 Endpoint (90 d) mean*: 350 μg/w: 96 2000 μg/w: 122	Low

* Estimated from figure by Graphreader.com.

** Converted from ng/mL.

### Risk of bias and strength of evidence

Overall risk of bias was considered *low* for Del Bo et al. (37) and Kwok et al. (34), *some concerns* for Lederer et al. (36) and *high* for Obersby et al. (35) (Fig. 3).

The strength of evidence that habitual B12 intake or an intake in line with the current Nordic RI is sufficient to ensure adequate status among vegetarians and/or vegans is considered *Limited – no conclusion* as there were no eligible prospective cohort studies that investigated B12 intake in relation to status among vegetarians or vegans.

## Discussion

The results of this systematic review show that there is a scarcity of prospective studies into B12 intake in relation to B12 status for most of the included populations. In addition, most intervention studies used high supplemental doses and short study durations, making it difficult to conclude on the long-term effect of low-dose B12 supplementation. There are some indications that breastfed infants have lower B12 status than non-breastfed infants. Data are however not sufficient to assess the relevance of this finding. There were not enough data to assess B12 intake in relation to B12 status in other groups.

In pregnancy, the strength of evidence that habitual B12 intake or an intake in line with the current Nordic RI is sufficient to ensure adequate status was considered *Limited – no conclusion*. Assessing dietary intake and status in the pregnant state is associated with some difficulties. A dietary assessment in early pregnancy can be obscured by pregnancy nausea and/or vomiting (38) and might not be an accurate reflection of habitual pre-pregnancy intake or intake as the pregnancy advances. In addition, plasma volume expansion can make changes in nutritional biomarkers over pregnancy difficult to assess (39). Future studies, preferably prospective- and population-based, are required to investigate how B12 intake relates to both maternal and infant B12 status.

During lactation, the strength of evidence that habitual B12 intake or an intake in line with the current Nordic RI is sufficient to ensure adequate status was considered *Limited – no conclusion*. There was however *Limited* – *suggestive* evidence that breastfed infants had lower B12 status than non-breastfed infants at 4–6 months of age. This is similar with findings from a 2017 systematic review that found insufficient evidence to evaluate the timing of introducing of complementary food or beverage on infant B12 status (40). Further, a 2018 systematic review found evidence for associations between breastmilk B12 content and maternal intake of B12 (9) but methodological disparities obscured any firm conclusions. Since infant B12 stores can be low if maternal intake during pregnancy was low, ensuring adequate B12 intake during lactation is important to prevent deficiency (41). The relevance of the observed lower B12 status among breastfed infants cannot be determined based on the findings included in the current review.

Among older adults, the strength of evidence that habitual B12 intake or an intake in line with the current Nordic RI is sufficient to ensure adequate status among older adults is considered *Limited – no conclusion*. The interventional studies overall found higher B12 status with higher B12 intake, but the definitions of what constituted optimal B12 status differed and thus the interpretations of the findings. The RCTs mostly included older adults with B12 deficiency, likely caused by reduced absorption (42), and it is difficult to transfer the results to maintenance of vitamin B12 status. Thus, the results should be interpreted with that in mind. In addition, almost all studies were short term (<6 months) and may have not shown the full potential of low-dose vitamin B12 supplements to normalize B12 status. It is estimated that B12 deficiency affects approximately 2%–35% of the older population, depending on age range and region (4). These estimates may not be representative of the general older adult population in the Nordic region. Population-based studies that assess the prevalence of B12 deficiency in relation to intake among older adults are warranted.

The strength of evidence that habitual B12 intake is sufficient to ensure adequate status among vegetarians and/or vegans is considered *Limited – no conclusion*. It is known that a vegan diet does not provide enough B12 and eventually requires supplementation (43). However, there was an unexpected lack of prospective cohort studies to show the effect of lacto-ovo vegetarian diet on B12 status. Thus, more prospective studies are needed to clarify if B12 supplementation should be recommended also to lacto-ovo vegetarians.

### Limitations and strengths

Strengths of this review include a highly standardized process of literature searches, article selection, data extraction, risk of bias assessment and evidence grading. Limitations of this work are mainly related to the scarcity of data and relevant studies. Since B12 deficiency takes years to develop; cross-sectional studies were only included for pregnant and lactating women. This is both a limitation and a strength, since it not only greatly reduced the number of eligible studies but also ensured higher quality data. Lastly, all included RCTs were short term and will thus not show the long-term effects of B12 supplementation.

## Conclusion

In conclusion, evidence is insufficient to assess if habitual B12 intake or an intake in line with the current Nordic RI is sufficient to maintain adequate status for all included populations. Population-based cohort studies and low-to-moderate dose interventions that address this question are highly warranted.
